# eTWST: An Extension to the Timed Water Swallow Test for Increased Dysphagia Screening Accuracy

**DOI:** 10.1007/s00455-024-10778-z

**Published:** 2024-11-09

**Authors:** Louise Brage, Fredrik Nylén, Patricia Hägglund, Thorbjörn Holmlund

**Affiliations:** 1 Speech and Language Pathology Department of Clinical Sciences, Umeå University, 90187 Umeå, Sweden; 2 Otorhinolaryngology, Department of Clinical Sciences, Umeå University, 90187 Umeå, Sweden

**Keywords:** Deglutition, Dysphagia screening, Water swallow test, Flexible endoscopic evaluationof swallowing

## Abstract

We aimed to fine-tuning the Timed Water Swallow Test (TWST) screening procedure to provide the most reliable prediction of the Flexible Endoscopic Evaluation of Swallowing (FEES) assessment outcomes, with age, sex, and the presence of clinical signs of dysphagia being considered in the assessment. Participants were healthy people and patients with suspected dysphagia. TWST performance and participants’ reported dysphagia symptoms were assessed in terms of their utility in predicting the outcome of a FEES assessment the same day. The FEES assessors were blinded to the nature of the TWST performance. The water swallowing capacity levels and clinical observations during a screening performance that were indicative of dysphagia/no symptoms in FEES were determined. Convergent validity was assessed as the agreement with the Functional Oral Intake Scale (FOIS) in the FEES assessment. TWST predicted FEES findings (aspiration and dysphagia) with a sensitivity of 72 and 45% and a specificity of 75% and 80%, respectively. Extended analysis of the TWST procedure (eTWST) identified aspiration (sensitivity = 92%, specificity = 62%) and dysphagia (sensitivity = 70%, and specificity = 72%) more accurately and showed a high correlation with FOIS (ɸ = 0.37). Excellent inter-rater reliability was further observed (Kw = 0.83). The extended evaluation of TWST performance has superior criterion validity to that of TWST. eTWST displayed high convergent validity and excellent interrater reliability. We therefore believe that eTWST can be highly relevant for clinical dysphagia screening.

## Introduction

Dysphagia affects 2–16% of the population, 36% of patients in hospital settings, and 50% of nursing care residents [1–4] and may have several health-threatening complications, such as aspiration pneumonia, malnutrition, dehydration, prolonged hospital stay [1–6], and death [7–10]. Despite its potential severe consequences, dysphagia is often undetected, and most patients with the condition are neither diagnosed nor treated [1, 11]. Early detection of dysphagia might lead to better care and quality of life for individuals with dysphagia and to reduced healthcare costs [4, 12–14]. Flexible Endoscopic Evaluation of Swallowing (FEES) and Videofluoroscopic Examination of Swallowing (VFS) are the gold standards for identifying dysphagia [15]. However, the requirement for specialized equipment and trained personnel prevents broad application aimed at identifying persons at risk of experiencing health consequences. Screening tools are designed for detecting patients who are at risk of dysphagia, and procedures with appropriate predictive accuracy in predicting subsequent diagnosis of dysphagia in VFS or FEES can have multiple benefits, such as reducing morbidity and mortality. Today, many screening tests for dysphagia are available, with varying sensitivity and specificity [4, 16–18]. For example, the 3-oz Water Swallow Test [19] and the more comprehensive multiple consistency test, the Gugging Swallowing Screen [20–22], have sensitivities of 76% and 59% and specificities of 81–100% and 59–79%, respectively, compared with a gold standard method. There is a need for a water swallowing test with more balanced prediction accuracy to support early but resource-friendly identification of persons at high risk of dysphagia in low-resource care contexts for subsequent referral to evaluation using VFS or FEES.

The Timed Water Swallow Test (TWST) [23] is a water swallowing capacity test designed to identify patients at risk of dysphagia. The test has been shown to have high intra- and inter-rater and test–retest reliabilities and to permit reliable administration via telemedicine [24, 25]. The patient is asked to drink a glass of water (150 mL) as quickly but safely as possible. The original instructions required the observation of coughing following the procedure but provided no guidance on how the observation should influence the outcome. A swallowing capacity of < 10 mL/s is currently used clinically to indicate dysphagia, based on initial validation against a clinical swallowing examination in patients < 70 years old [23] and among acute stroke patients [26]. A swallowing capacity of < 10 mL/s has, in subsequent studies, been concluded to indicate a swallowing dysfunction according to VFS [27, 28]. Several studies have noted that water swallowing capacity differs between the sexes [29–31] and that advancing age also has a strong impact on water swallowing capacity [1, 2, 4, 31, 32]. Further clinical observations have led us to suspect that a water swallowing capacity < 10 mL/s may be too forgiving. The purpose of this investigation was to re-evaluate the assessment of TWST performance to provide the most reliable prediction of the outcome compared with FEES evaluation of the same participant, with age, sex, and the presence of clinical signs of dysphagia being considered in the assessment.

## Materials and Methods

### Study Design

This prospective validation study of TWST against FEES was approved by the Swedish Ethical Review Authority (Dnr 2020–04817, 2022–06021-02, 2023–05938-02) and conducted in accordance with the STARD guidelines [33] (see Appendix A) and the Helsinki Declaration [34].

## Participants

Eligible participants in the current study were (a) healthy people aged ≥ 20 years with no history of dysphagia or diagnosis related to dysphagia and (b) patients with suspected dysphagia (e.g., due to stroke, Parkinson’s disease, or after anterior cervical spine surgery) admitted to the University Hospital of Umeå. All participants gave their written informed consent before inclusion.

## Procedure

The participants completed TWST and FEES within two hours, with the total examination lasting around 30 min. To permit determination of inter-rater reliability, the TWST procedure was recorded using two cameras (Canon EOS 1100D and Sony FDR-AX43A). The angle of the video recording was from the tip of the participant’s nose down toward the collarbone. The FEES assessment was carried out using a video endoscope (model ENF-VH, Olympus). Clinical data were collected for each participant, covering age, sex, height, weight, BMI, and dysphagia sensation.

## TWST Assessment

Before the TWST procedure, which were performed by experienced speech-language pathologists (SLPs), the participants ingested three teaspoons of water to ensure no overt signs of aspiration. If any signs of aspiration occurred (e.g., coughing or wet voice), a 0 mL/s swallowing capacity was noted during TWST, and no further assessments were performed. For participants who could safely swallow water, the TWST assessment proceeded, and they were asked to ingest 150 mL of water as quickly as possible but to stop if they experienced any difficulties [23, 29]. After completing the ingestion, the participants were instructed to produce a prolonged vowel “a” to permit the observation of a wet or gurgly voice after testing. The time, in seconds, taken to swallow the water was recorded from when the glass of water touched the lips to when the participant began producing the vowel, or when the larynx returned to a resting position. In addition, the number of swallows and any residual water (mL) were observed. Swallowing capacity (mL/s) was calculated by dividing the volume of water swallowed, with residual liquid subtracted from the 150 mL provided, by the time taken to ingest the water. Additional clinical signs of dysphagia, such as coughing, wet or gurgly voice, or nasal regurgitation, were also noted.

## FEES Assessment

FEES was used as the reference standard for validating TWST as it is considered (a) the gold standard for detecting dysphagia and (b) can be performed bedside [35–37]. Two experienced speech-language pathologists (SLPs) performed the FEES assessment in accordance with the protocol described by Langmore et al. [38]. The SLPs who performed the FEES were blinded to the participant’s TWST results. FEES was performed to visualize the hypopharynx and larynx and to assess the safety and efficacy of swallowing. Four bolus consistencies were tested during FEES: levels 0, 2, 4, and 7 according to the International Dysphagia Diet Standardisation Initiative (IDDSI) framework [39]. The bolus was dyed with green food coloring to enable visibility. Three teaspoons and three tablespoons of mildly thick (IDDSI 2), liquid (IDDSI 0), and extremely thick (IDDSI 4) substances were given in consecutive order. The patient was also instructed to drink two or three mouthfuls of liquid (IDDSI 0) and to masticate and swallow a piece of cracker (IDDSI 7). The protocol was modified or shortened if the SLP determined that the patient’s safety was at risk due to significant aspiration or other severe dysphagia symptoms.

Based on the FEES findings, the presence of dysphagia was classified according to the Dysphagia Outcome and Severity Scale (DOSS) [40]. A DOSS score of 5 or less was defined as indicating the presence of dysphagia. Aspiration risk was classified according to the Penetration–Aspiration Scale (PAS) [41], with a score > 4 indicating aspiration risk. Based on the FEES assessment, the examiner also rated the patient’s ability regarding food and drink intake according to the Functional Oral Intake Scale (FOIS) [9], as a basis for assessing convergent validity.

## Statistical Analysis

The criterion validity of TWST was determined using the clinical determination of dysphagia in FEES (DOSS of ≤ 5 and PAS > 4 indicating dysphagia) as the description to be predicted. The water swallowing capacity observed for the participant was assessed following the standard TWST guidelines so that a swallowing capacity of < 10 mL/s was considered a prediction of dysphagia by the TWST screening, and a swallowing capacity of ≥ 10 mL/s indicated no symptoms of dysphagia. The level of agreement between the TWST prediction and FEES outcome for the participant then determined the TWST screening accuracy. For each measure of the TWST screening’s ability to predict the FEES assessment outcome, 95% confidence intervals were computed from 1000 bootstrap resamples of the dataset.

Subsequently, the expanded evaluation of possible optimizations of the TWST screening was explored based on the measured water swallowing capacity, observations of clinical signs of dysphagia during the TWST performance, and the age and sex of the participants. An iterative process using statistical decision trees to model the FEES binary outcome (dysphagia or no symptoms of dysphagia) was entered using a range of tree depths and parameters to determine important cutoff points for each participant’s FEES assessment. The aim was to identify the most efficient predictors of FEES outcomes given what can be observed during TWST performance, with idiosyncratic patterns arising due to one or a few participants being factored out. The resulting extended version of TWST (eTWST) was assessed in terms of its ability to predict a DOSS score ≤ 5 or PAS score > 4 in FEES from the new eTWST with a sensitivity, specificity, and overall accuracy of at least 70%. The 95% confidence intervals for all measures of the eTWST’s ability to predict the FEES outcomes were computed on the same bootstrap resampled datasets used when evaluating TWST.

The convergent validity of both the TWST and eTWST screening methods was determined by comparing their outcomes (i.e., dysphagia or no symptoms of dysphagia) with the outcome of FOIS based on the comprehensive FEES assessment. The sensitivity, specificity, and overall accuracy of the screening method (i.e., TWST or eTWST) in predicting the FOIS score (FOIS ≤ 6 or FOIS of 7, respectively) were calculated. In addition, participant reports of swallowing difficulties in everyday life were assessed in terms of their agreement with the outcome of the FEES assessment.

Inter-rater agreement was estimated from two raters’ independent observations of TWST outcome measures from the video recordings. The recordings were selected for re-evaluation using a stratified random sampling procedure. In that procedure, age, sex, and participant group (healthy persons and patients with suspected dysphagia) were represented in proportion to their occurrence in the whole sample.

## Results

During the study period (January 2023–March 2024), a total of 198 people were eligible to participate; two participants were excluded due to not giving consent to be examined using FEES. An overview of the participants is presented in Table 1. Of the 196 participants, 84 were women and the median age was 66 years (range 21–91 years). No adverse events occurred during the assessments.

**Table 1:** Epidemiological and Subject Characteristics (*n* = 196)

Participants	196 (100)
Healthy adults	127 (65)
Age, median (range)	58 (21–91)
Sex	
Women	61 (48)
Men	66 (52)
Suspected dysphagia	69 (35)
Age, median (range)	71 (38–88)
Sex	
Women	23 (33)
Men	46 (67)
Etiology of dysphagia	
Stroke	10 (5)
Transient ischemic attack	1 (0.5)
Anterior cervical spine surgery	41 (21)
Head/neck cancer	3 (2)
Lewy body dementia	1 (0.5)
Spinocerebellar ataxia	1 (0.5)
Parkinson’s disease	7 (4)
Kennedy’s disease	1 (0.5)
Unknown cause	4 (2)
Entire group of participants	196 (100)
Age, median (range)	66 (21–91)
Sex	
Women	84 (43)
Men	112 (57)
BMI, median (range)	25 (17–41)

Data are given as number of participants, *n* (%), or median (IQR)

## TWST and FEES Assessments

In total, 55 (28%) participants were observed with a swallowing capacity < 10 mL/s, and 68 (35%) participants showed clinical signs of dysphagia during the TWST assessment. According to the FEES assessment, aspiration was observed in 13 (7%) of the 196 participants (PAS score > 4) and 61 (31%) participants were classified as having dysphagia (DOSS score ≤ 5).

Fifty-seven (29%) participants reported subjective swallowing problems in everyday life; of these, dysphagia was observed in 31 (54%) participants and aspiration in four (0.7%) according to the FEES assessment. Furthermore, of those participants with a sensation of dysphagia, 28 (49%) were observed with a swallowing capacity < 10 mL/s, and 35 (61%) participants showed clinical signs of dysphagia during the TWST assessment. An overview of the participants’ responses regarding subjective dysphagia is presented in Table 2.

**Table 2:** Overview of Participants’ Responses Regarding Subjective Dysphagia (*n* = 196)

Sensation of dysphagia	57 (29)
Classified as having dysphagia (DOSS score ≤ 5 or PAS > 4)	31 (54)
Observed aspiration (PAS > 4)	4 (0.7)
Swallowing capacity < 10 mL/s	28 (49)
Clinical signs of dysphagia during TWST	35 (61)

Data are given as number of participants, *n* (%) *DOSS* Dysphagia Outcome Severity Scale, *PAS* Penetration–Aspiration Scale, *TWST* Timed Water Swallow Test

## Criterion Validity: TWST

With a cut-off value of 10 mL/s, as per the original clinical validation of TWST, the test identified aspiration with a sensitivity of 72% [CI_95%_ = 0.55–0.93] and a specificity of 75% [CI_95%_ = 0.69–0.80]. With the same cut-off, 10 mL/s, the test identified dysphagia with a sensitivity of 45% [CI_95%_ = 0.33–0.53] and a specificity of 80% [CI_95%_ = 0.74–0.85]. An overview of the sensitivity, specificity, and predictive values of TWST in detecting dysphagia, as determined using the FEES assessment scales, is presented in Table 3.

**Table 3:** Sensitivity, Specificity, and Predictive Values in the Timed Water Swallow Test in Detecting Dysphagia, as Determined by the Gold Standard FEES Assessment and the PAS and DOSS Evaluation Scales (*n* = 196)

	FEES Results		
	Aspiration Risk (PAS 5–8)	No Aspiration Risk (PAS 1–4)	
TWST results			
Swallowing capacity < 10 mL/s	9	46	PPV = 16% [0.08–0.24]
Swallowing capacity ≥ 10 mL/s	4	137	NPV = 97% [0.95–0.99]
Sensitivity	72% [0.55–0.93]	Specificity	75% [0.69–0.80]
Accuracy	75% [0.69–0.80]	Prevalence	6% [0.03–0.09]

	FEES Results		
	Dysphagia (DOSS 1–5 or PAS 5–8)	Normal Swallowing (DOSS 6–7 or PAS 1–4)	
TWST results			
Swallowing capacity < 10 mL/s	28	27	PPV = 50% [0.41–0.65]
Swallowing capacity ≥ 10 mL/s	33	108	NPV = 76% [0.72–0.83]
Sensitivity	45% [0.33–0.53]	Specificity	80% [0.74–0.85]
Accuracy	69% [0.64–0.74]	Prevalence	31% [0.26–0.38]

*FEES* Flexible Endoscopic Evaluation of Swallowing, *TWST* Timed Water Swallow Test, *PAS* Penetration–Aspiration Scale, *PPV* Positive predictive value, *NPV* Negative predictive value. Prevalence: the proportion of participants with dysphagia according to the FEES evaluation. Results are presented descriptively or as percentages [95% CI]

## Criterion Validity: eTWST

**Fig. 1:**
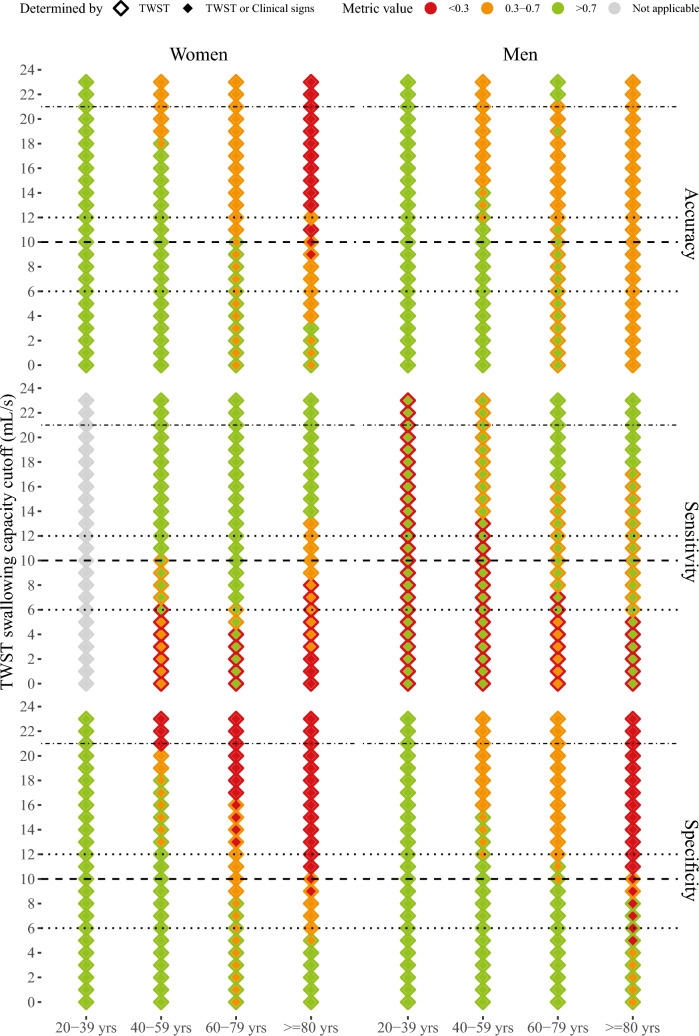
Sex- and age-stratified sensitivity, specificity, and overall accuracy in predicting the FEES (DOSS ≤ 5 or PAS > 4) outcome from TWST performance but with varying swallowing capacity cutoff values used. The figure further displays the effect of additionally considering the observations of clinical signs of dysphagia when a certain swallowing capacity cutoff has been employed

**Fig. 2:**
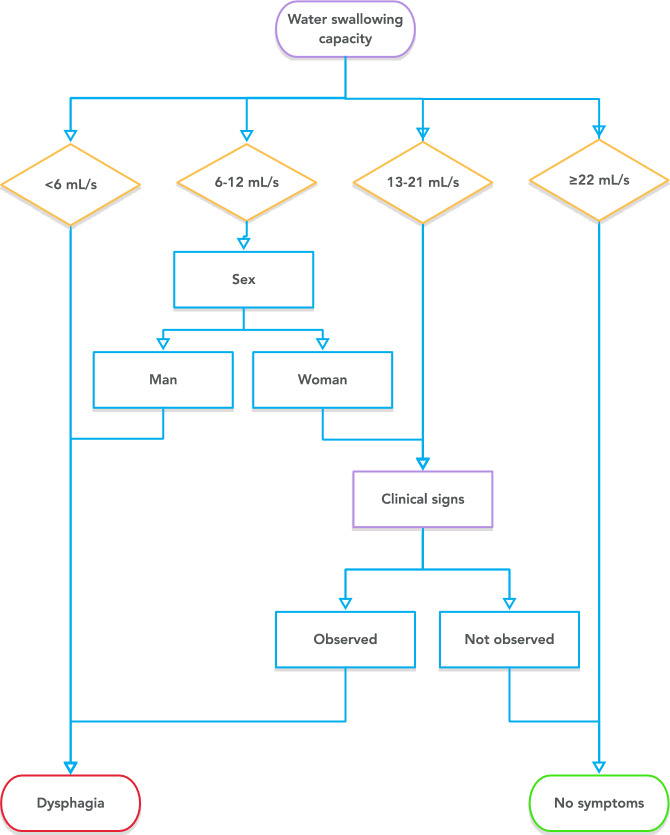
The eTWST screening evaluation procedure

Sex- and age-stratified sensitivity, specificity, and overall accuracy in predicting the FEES (DOSS ≤ 5 or PAS > 4) outcome from TWST performance but with varying swallowing capacity cutoff values used is presented in Fig. 1. The figure further displays the effect of additionally considering the observations of clinical signs of dysphagia when a certain swallowing capacity cutoff has been employed. The eTWST screening evaluation procedure (Fig. 2) identified aspiration with a sensitivity of 92% [CI_95%_ = 0.80–1.00] and a specificity of 62% [CI_95%_ = 0.58–0.68]. eTWST further identified dysphagia with a sensitivity of 70% [CI_95%_ = 0.60–0.76] and a specificity of 72% [CI_95%_ = 0.66–0.70]. An overview of the sensitivity, specificity, and predictive values of eTWST in detecting dysphagia, as determined using the FEES assessment scales, is presented in Table 4.

**Table 4:** Sensitivity, Specificity, and Predictive Values in the Extended Timed Water Swallow Test in Detecting Dysphagia as Determined by the Gold Standard FEES Assessment and the PAS and DOSS Evaluation Scales (*n* = 196)

	FEES Results		
	Aspiration Risk (PAS 5–8)	No Aspiration Risk (PAS 1–4)	
eTWST results			
Dysphagia^*^	12	68	PPV = 14% [0.08–0.20]
No dysphagia	1	115	NPV = 99% [0.98–1.00]
Sensitivity	92% [0.80–1.00]	Specificity	62% [0.58–0.68]
Accuracy	64% [0.60–0.70]	Prevalence	6% [0.03–0.09]

	FEES Results		
	Dysphagia (DOSS 1–5 or PAS 5–8)	Normal Swallowing (DOSS 6–7 or PAS 1–4)	
eTWST results			
Dysphagia	43	37	PPV = 53% [0.44–0.65]
No dysphagia^*^	18	98	NPV = 84% [0.78–0.89]
Sensitivity	70% [0.60–0.76]	Specificity	72% [0.66–0.76]
Accuracy	71% [0.66–0.76]	Prevalence	31% [0.26–0.38]

*FEES* Flexible Endoscopic Evaluation of Swallowing, *eTWST* extended Timed Water Swallow Test, *PAS* Penetration–Aspiration Scale, *PPV* positive predictive value, *NPV* negative predictive value. Prevalence: the proportion of participants with dysphagia according to the FEES evaluation. Results are presented descriptively or as percentages [95% CI].* In eTWST, dysphagia was determined based on the following: (a) swallowing capacity < 6 mL/s (women) and < 13 mL/s (men) or (b) for the intermediate range (6–21 mL/s for women and 13–21 mL/s for men), the observation of clinical signs determined the presence of dysphagia

## Convergent Validity: eTWST

The eTWST assessment showed a high correlation with the FOIS (ɸ = 0.37 [CI_95%_ = 0.22–0.51]; *r* = 0.37 [CI_95%_ = 0.25–0.49], *p* < 0.001); the correlation between FOIS and the original TWST was lower although statistically significant (ɸ = 0.27 [CI_95%_ = 0.12–0.42]; *r* = 0.28 [CI_95%_ = 0.14–0.41], *p* < 0.001).

## Inter-rater Reliability

Forty participants’ (20%) TWST assessments were analyzed twice by two SLPs. The inter-rater reliability between the two raters regarding clinical signs of dysphagia showed strength of agreement (_Kw_ = 0.83 [CI_95%_ = 0.65–1.00], *p* ≤ 0.001) as computed using Cohen’s weighted kappa with quadratic weights. The inter-rater reliabilities regarding the time taken to swallow the water and the number of swallows were computed using intra-class correlation coefficients and showed strength of agreement (ICC = 1.00 [CI_95%_ = 0.99–1.00]; ICC = 0.99 [CI_95%_ = 0.98–0.99], respectively).

## Discussion

This study related participants’ performances in the TWST screening procedure to the dysphagia status of the participant, as established in a FEES assessment conducted the same day. We included 196 participants, both healthy participants as well as patients with a condition that mandated a FEES assessment for dysphagia, of both sexes and aged 21–91 years. The FEES assessment was performed with the clinical evaluator being blinded as to the screening outcome. The findings indicate that the high criterion and convergent validities of a TWST performance may be considerably enhanced by extending the evaluation procedure.

For criterion validity, TWST was shown to afford a sensitivity of 92% and specificity of 62% in identifying aspiration, and a sensitivity of 70%, specificity of 72%, and overall accuracy of 71% in predicting dysphagia as per the comprehensive FEES assessment. This level of screening accuracy is achieved by augmenting the TWST performance by (a) including observed clinical signs of dysphagia during or following the test and (b) using a sex-dependent swallowing capacity level (6 mL/s for women and 13 mL/s for men) below which dysphagia is indicated. High inter-rater reliability was demonstrated for the observations required to perform the screening (water swallowing capacity and clinical symptoms of a swallowing dysfunction). We propose that the extended TWST should be called eTWST to avoid confusion. The eTWST evaluation was shown to be superior to the original TWST in our sample; TWST results evaluated using the 10 mL/s criterion alone achieved a sensitivity of 45%, specificity of 80%, and overall accuracy of 69% in predicting the FEES assessment outcome for our participants. Thus, while eTWST met the minimum criteria in terms of sensitivity and specificity for dysphagia diagnosis (70% and 60%, respectively) [42], the original manner of evaluating a TWST performance did not. Our data indicate that a patient’s reported level of swallowing problems in everyday life is not a reliable indicator of the actual presence of dysphagia.

Our study is not the first to observe the relevance of clinical signs of swallowing dysfunction after having swallowed water under time constraints. Coughing or altered voice quality was considered in the development of TWST [29], but only noted and not formalized as part of evaluating the screening procedure. Our conclusion that clinical signs should be included in the evaluation is given external support by some more recently published works. Brodsky et al. concluded, based on their meta-analysis [43], that consecutive sips of large volumes of water in patients with no overt airway responses or voice changes appropriately ruled out the risk of aspiration. Kuuskoski et al. concluded in their evaluation of the predictive performance of the Water Swallowing Test (WST) that “coughing and average drinking bolus size are the most important parameters in WST when screening for referral to VFS, whereas the swallowing speed does not seem to be useful” [30]. Our data instead suggest that the predictive ability of TWST is considerably enhanced if the observation of clinical signs is incorporated into the evaluation of the screening procedure. Clear cases can be determined primarily based on the swallowing capacity achieved: a swallowing capacity below < 6 mL/s (women) and < 13 mL/s (men) indicates dysphagia, whereas ≥ 22 mL/s indicates no symptoms of dysphagia. In intermediate cases, a swallowing capacity of 6–21 mL/s for women and 13–21 mL/s for men, we observed that the absence or presence of clinical signs is highly indicative as a determinant of the screening procedure outcome. Compared with other water swallowing tests, eTWST offers better accuracy in predicting dysphagia and high sensitivity in detecting aspiration during FEES. It also shows strong agreement with FOIS results, supporting its validity as a dysphagia screening tool.

In line with previous findings, we observed a sex difference in swallowing capacity, with women having lower capacity than men. This difference may become more pronounced with age [1, 2, 4, 31, 32]. Our study included participants aged 21–91 years, with a balanced control group. However, older participants with swallowing disorders were more prevalent in our samples than were those without dysphagia. eTWST was, however, indicated to be more predictive than the original TWST evaluation based on a 10 mL/s cutoff, and may therefore potentially be less reliably transferred to other samples of participants of advanced ages. In our evaluation, the FEES outcomes were used as the reference test and ground truth with which the water swallowing performance was compared. We did not, however, conduct an intra- or inter-rater evaluation of the FEES assessment. FEES was, however, performed by specially trained and experienced clinical evaluators, in agreement with standardized clinical practice. Thus, while verification of the reliability of the FEES comparison could have been incorporated into the design of this study, we consider the findings reliable and the eTWST evaluation shown to provide an enhanced screening procedure compared with the original TWST.

## Conclusion

The extended evaluation of TWST performance has better criterion validity than does the original TWST. As eTWST showed high convergent validity and excellent inter-rater reliability, we believe that it can be highly relevant in the clinical setting for dysphagia screening.

## Data Availability

Data supporting the results and analyses are available on request from the final author (TH).
